# RB, p130 and p107 differentially repress G1/S and G2/M genes after p53 activation

**DOI:** 10.1093/nar/gkz961

**Published:** 2019-10-31

**Authors:** Amy E Schade, Martin Fischer, James A DeCaprio

**Affiliations:** 1 Program in Virology, Division of Medical Sciences, Graduate School of Arts and Sciences, Harvard University, Boston, MA 02115, USA; 2 Department of Medical Oncology, Dana-Farber Cancer Institute, Boston, MA 02215, USA; 3 Computational Biology Group, Leibniz Institute on Aging - Fritz Lipmann Institute (FLI), 07745, Jena, Germany; 4 Department of Medicine, Brigham and Women's Hospital and Harvard Medical School, Boston, MA 02115, USA

## Abstract

Cell cycle gene expression occurs in two waves. The G1/S genes encode factors required for DNA synthesis and the G2/M genes contribute to mitosis. The Retinoblastoma protein (RB) and DREAM complex (DP, RB-like, E2F4 and MuvB) cooperate to repress all cell cycle genes during G1 and inhibit entry into the cell cycle. DNA damage activates p53 leading to increased levels of p21 and inhibition of cell cycle progression. Whether the G1/S and G2/M genes are differentially repressed by RB and the RB-like proteins p130 and p107 in response to DNA damage is not known. We performed gene expression profiling of primary human fibroblasts upon DNA damage and assessed the effects on G1/S and G2/M genes. Upon p53 activation, p130 and RB cooperated to repress the G1/S genes. In addition, in the absence of RB and p130, p107 contributed to repression of G1/S genes. In contrast, G2/M genes were repressed by p130 and p107 after p53 activation. Furthermore, repression of G2/M genes by p107 and p130 led to reduced entry into mitosis. Our data demonstrates specific roles for RB, p130-DREAM, and p107-DREAM in p53 and p21 mediated repression of cell cycle genes.

## INTRODUCTION

In response to DNA damage, cells can slow cell cycle progression to enable DNA repair. Repression of cell cycle regulated genes during the DNA damage response contributes to the proliferation arrest. Loss of the tumor suppressor proteins RB and p53 perturb the response of the cell cycle genes to DNA damage in cancer (1). Importantly, DNA damaging agents such as doxorubicin are commonly used as chemotherapeutics in a variety of cancer types (2). Understanding how cell cycle gene expression is repressed in response to DNA damage can provide significant insight into how cancer cells respond to chemotherapy.

Cell cycle gene expression occurs in two distinct waves (3). The early or G1/S wave begins during late G1 and consists of genes encoding factors required for DNA replication. G1/S genes are regulated by the E2F transcription factors that bind to E2F DNA elements in their promoters (4). Activator E2Fs (E2F1, E2F2, and E2F3a) and their dimerization partners (DP1 and DP2) transactivate G1/S cell cycle genes during late G1, enabling cells to pass through the restriction point and enter into S phase (5–8). The RB protein, encoded by the *RB1* retinoblastoma tumor suppressor gene, binds to the activator E2Fs during G0 and G1 and represses G1/S genes (9). In addition to RB, the RB-like protein p130 (encoded by the *RBL2* gene) binds the repressor E2F4 and the MuvB (multivulva class B) core consisting of LIN9, LIN37, LIN52, LIN54 and RBBP4 to form the DREAM complex (DP, RB-like, E2F and MuvB) (10–12). The repressor E2F4 component of DREAM binds to the E2F sites in the G1/S gene promoters (11,13,14). The DREAM complex and RB cooperate to repress G1/S gene expression during G0 (13,15).

The late or G2/M wave of cell cycle genes encodes factors required for mitosis(3,5). During G0 and G1, the DREAM complex binds and represses G2/M genes by MuvB specific binding to CHR elements present in their promoters (11,13–15). During S/G2, the MuvB core sequentially recruits BMYB (*MYBL2*) and then FOXM1 to CHR elements in G2/M gene promoters (16–19). G2/M genes are transactivated by the MYB-MuvB (MMB)-FOXM1 complex (20) upon activation by CDK phosphorylation (10,20,21).

Relief of repression by RB and DREAM of G1/S gene expression during cell cycle entry is dependent, at least in part, on Cyclin-CDK phosphorylation of p130 and RB. During G1, RB is phosphorylated by Cyclin D-CDK4/6 and Cyclin E-CDK2 (13,22,23). Cyclin D-CDK4/6 phosphorylation of p130 during mid-G1 results in DREAM complex disruption in a two step process when phosphorylated p130 and MuvB are released together from chromatin followed by separation of p130 from MuvB and E2F4 release from chromatin (13).

The p107 protein (encoded by the *RBL1* gene) is structurally similar to p130 and has been reported to bind repressor E2F and MuvB in some cell lines (12,22,24). *RBL1* itself is a G1/S gene and is expressed during S phase (25,25). When p107 functions as a cell cycle gene repressor remains unclear as it is phosphorylated and presumably inactivated by Cyclin-CDK complexes in proliferating cells (26,27). We recently reported that, in the absence of p130, p107 can form a DREAM-like complex containing either p107-E2F4 or p107-MuvB during G0 (13). Whether p107 forms a functional transcriptional repressor complex and when it functions remains an open question.

In response to DNA damage, the tumor suppressor protein p53 (*TP53*) is activated and contributes to cell cycle arrest (reviewed in (28)). p53 mediated down-regulation of cell cycle genes after DNA damage is well conserved in human and mouse cells and reflects a key role for p53 in cell cycle arrest (29). Emerging evidence indicates that p53 acts as an activator of gene expression and indirectly represses cell cycle gene expression (5,30). Cellular stresses that induce DNA damage can block cell cycle progression at the G1/S checkpoint. DNA damage activation of p53 leads to increased levels of the CDK inhibitor p21 (*CDKN1A*) that, in turn, can inhibit Cyclin E-CDK2 and Cyclin A-CDK2 kinase activity (31,32). The DREAM complex is stabilized and bound to the G1/S and G2/M gene promoters when p21 is activated by p53 (33–35). In addition, RB contributes to p53 dependent down-regulation of many cell cycle genes (36–41). Whether DREAM and RB cooperate to repress G1/S and G2/M genes in response to p53 activation of p21 is not known. Here, we sought to determine the specific roles of p130, p107, RB and the DREAM complex in regulating G1/S and G2/M cell cycle gene levels upon DNA damage.

## MATERIALS AND METHODS

### Cell culture

HFFs were grown in DMEM supplemented with 15% fetal bovine serum, 1% Pen/Strep (Gibco), and 1% GlutaMAX (Gibco) at 37°C in 5% CO_2_. SaOS-2 cells with inducible p21 were a gift from Panagiotis Galanos and grown in DMEM supplemented with 10% fetal bovine serum, 1% Pen/Strep and 1% GlutaMAX (44). Where indicated, cells were treated with 350 nM doxorubicin (Cell Signaling), 250 nM RG7388 or 1 μg/ml doxycycline (Sigma-Aldrich).

### RNA sequencing and analysis

Total RNA was isolated from HFFs using Qiagen RNeasy plus kit, treated with DNase (Turbo DNA-free, Life Technologies), and quality control checked using Qubit, TapeStation, and qPCR. Libraries were prepared using Kappa stranded mRNA Hyper Prep and single-end sequenced using Illumina NS500. SaOS-2 total RNA was isolated using Qiagen RNAeasy plus kit, quality checked and quantified using the Agilent Bioanalyzer 2100 in combination with the RNA 6000 Nano Kit (Agilent Technologies). Libraries were constructed from 500 ng of total RNA using TruSeq stranded mRNA Library Preparation Kit (Illumina) following the manufacturer's description. Quantification and quality check of cDNA libraries was performed using the Agilent Bioanalyzer 2100 in combination with the DNA 7500 Kit. Libraries were pooled and sequenced in one lane on a HiSeq2500 in 51 cycle/single-end/high-output mode. Reads were mapped to the Hg19 genome by STAR (57). HTseq was used to generate count file with gene names (58). R package *DESeq2* was used to normalize counts (mean-ratio method), calculate total reads, and determine differential gene expression (59). MA plots were generated to display differentially expressed genes. Principal component analysis showed sufficient clustering of biological replicates. Volcano plots were generated using R package *EnhancedVolcano* (60). Heatmaps were generated using R package *ggplot2* function heatmap.2 (61).

### Cell cycle gene lists

Predicted G1/S and G2/M genes were extracted from an earlier meta-analysis (5). A list of ‘high-stringency’ G1/S and G2/M genes displayed peak expression in G1/S or G2/M respectively in at least three out of five cell cycle datasets. A list of ‘low stringency’ G1/S and G2/M genes displayed peak expression respectively in at least two out of five cell cycle datasets. Gene lists are reported in Supplemental Table S1.

### siRNA knockdown

Cells were transfected with 5 pmol siRNA using Lipofectamine RNAiMAX Transfection Reagent (Life Technologies). Specific siRNA oligos are reported in Supplemental Table S2.

### RT-qPCR

For RT-qPCR in HFF samples, total RNA was isolated using Qiagen RNeasy plus kit. cDNA was synthesized using High-Capacity reverse transcription kit (Thermo). qPCR was performed using Brilliant III SYBR Master Mix using Aria Mx3000 real time PCR machine (Agilent Genomics). Ct values were normalized to ACTB and B2M. For RT-qPCR in SaOS-2 p21 samples, RT-qPCR was performed on a Quantstudio 5 (ThermoFisher) using the Power SYBR Green RNA-to-CT 1-Step Kit (ThermoFisher). Ct values were normalized to U6. Primer sequences are reported in Supplemental Table S2.

### Cell cycle analysis

To measure S phase, cells were incubated with BrdU for 1 h before harvest, permeabilized with 2N HCl and blocked with Super Block Buffer (Thermo) in 0.1 M sodium borate for incubation with anti-BrdU-FITC antibody (Fisher Scientific) and propidium iodide with RNase I before analysis by flow cytometry (BD FACS Canto II). To measure mitosis, cells were harvested and fixed in 4% formaldehyde and then 70% cold ethanol. Cells were incubated with anti-phospho-H3-Alexa488 antibody (Cell Signaling) and propidium iodide with RNase I before flow cytometry.

### Protein analysis

Whole cell lysates were prepared in EBC lysis buffer, resolved by SDS-PAGE, and immunoblotted on nitrocellulose membranes as previously described (13). To preform immunoprecipitations, whole cell lysates were incubated with antibody and magnetic Protein A/C beads overnight at 4°C. Beads were washed 5× with EBC lysis buffer and analyzed by immunoblot. Antibodies are reported in Supplemental Table S2.

## RESULTS

### Cell cycle genes are deregulated after DNA damage in cells lacking RB and p130

**Figure 1.**
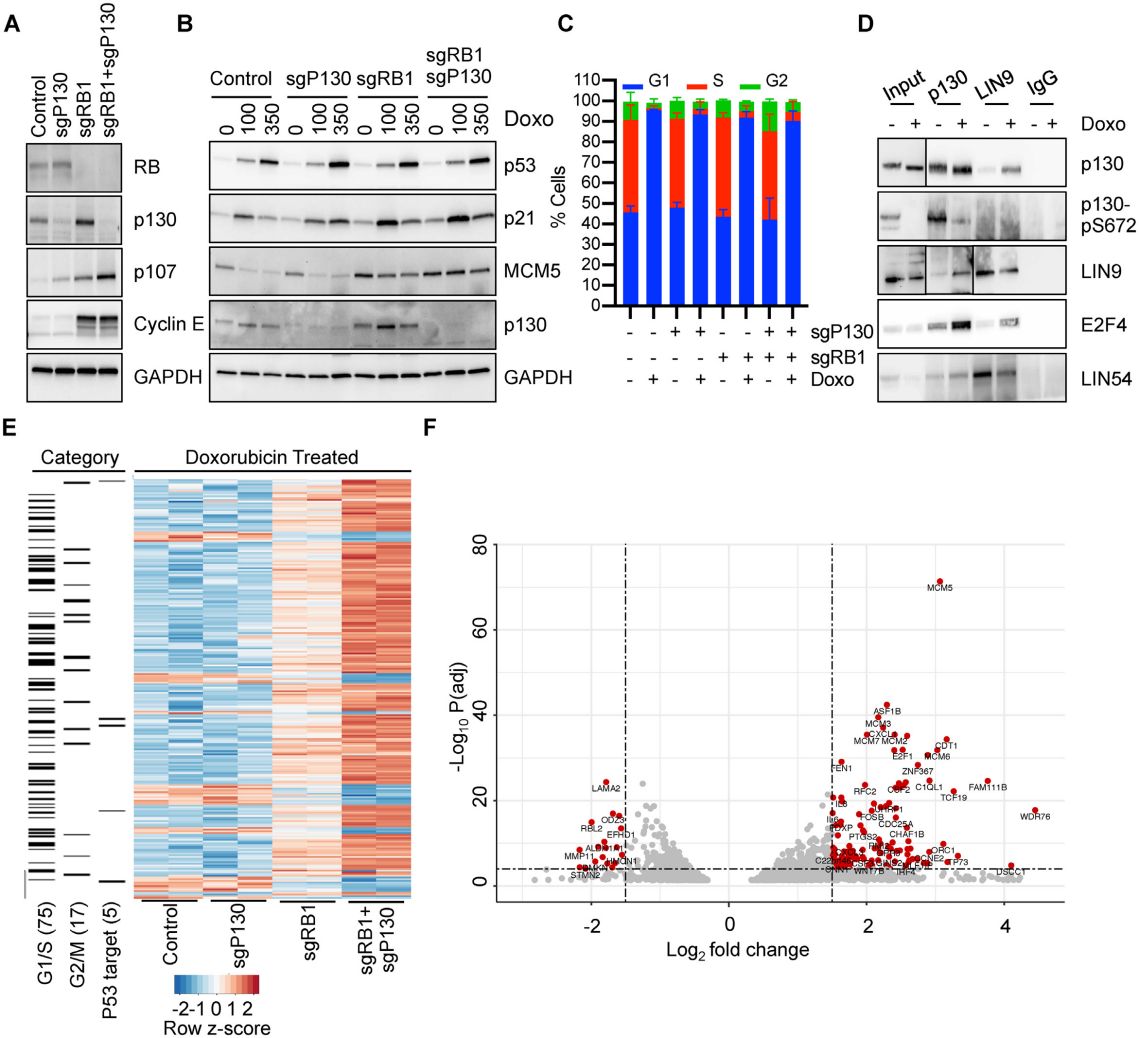
Cell Cycle genes are deregulated after DNA damage in cells lacking RB. (**A**) Immunoblot of HFFs with CRISPR-Cas9 mediated knockout of p130 and/or RB in contact arrest and serum starvation conditions. (**B**) Immunoblot of Control, sgP130, sgRB1, or sgRB1+sgP130 cells split into doxorubicin and harvested after 24 h. (**C**) Same as (**B**) but cell cycle phase was determined by BrdU incorporation and PI stain (*n* = 3). (**D**) Immunoblot of lysates prepared from p130, LIN9 and IgG immunoprecipitations using lysates from HFFs split into doxorubicin and harvested after 24 h. (**E**) RNA-seq of Control, sgP130, sgRB1, or sgRB1+sgP130 cells split into doxorubicin and harvested 24 h later (*n* = 2). Heatmap comparing top 250 differentially expressed genes amongst doxorubicin treated samples for each cell line with G1/S, G2/M or p53 target gene categorization indicated. (**F**) Volcano plot of differentially expressed genes from (**E**) with cut off of Log_2_ fold change of 1.5 and adjusted *P* value of 0.0001. See also Supplemental Figure S1.

To determine the roles of RB and p130 in response to DNA damage, we utilized primary HFFs with CRISPR-Cas9 mediated knockout of *RB1* (RB) or *RBL2* (p130) selected sequentially using puromycin (puro) and neomycin (neo) resistance cassettes and confirmed by next generation sequencing (13). RB and p130 were deleted alone or in combination to generate sgP130 (sgControl-puro, sgP130-neo), sgRB1 (sgRB1-puro, sgControl-neo), sgRB1+sgP130 (sgRB1-puro, sgP130-neo) and Control (sgControl-puro, sgControl-neo) cells. Reduced levels of p130 and RB protein were observed in the appropriate knockout lines by immunoblot of lysates prepared from contact-arrested, serum-starved, quiescent cells (Figure 1A). As previously reported, increased levels of p107 and Cyclin E, encoded by G1/S genes, were observed in the quiescent single knockout sgRB1 and double knockout sgRB1+sgP130 knockout cells compared to Control cells (42). Increased levels of p107 were also detected in the single knockout sgP130 cells (Figure 1A).

To determine if loss of p130 and RB affected levels of cell cycle genes after DNA damage, contact arrested knockout HFFs were split into subconfluent cultures with increasing concentrations of doxorubicin for 24 h before harvesting (Figure 1B). We observed an increase in p53 and p21 levels with doxorubicin treatment in each cell line, indicating that loss of p130 or RB did not affect p53 activation (Figure 1B). Notably, levels of MCM5, encoded by a G1/S gene, were similarly decreased in Control and sgP130 cells after doxorubicin but were not reduced in cells lacking RB alone or both RB and p130 (Figure 1B). As expected, untreated cultures harvested 24 h after release from contact arrest were enriched for cells in S phase (Figure 1C). Strikingly, doxorubicin resulted in a significant enrichment of cells in G1 and reduced levels in S phase in all four cell types despite persistent MCM5 expression in sgRB1 and sgRB1+sgP130 cells.

The DREAM complex is normally present in quiescent cells and is disrupted with release of p130 from MuvB and E2F4 upon entry into the cell cycle. To examine the status of the DREAM complex in cells undergoing DNA damage, lysates were prepared from parental HFFs with or without doxorubicin treatment after release from contact arrest for 24 h. Consistent with the disruption of the DREAM complex upon entry into S phase, untreated cells had low levels of co-immunoprecipitation of p130 with E2F4 and the MuvB components LIN9 and LIN54. In contrast, doxorubicin treated cells had increased levels of MuvB, E2F4, and p130 co-precipitation indicative of an intact DREAM complex. Consistent with this observation, doxorubicin treatment led to reduced levels of p130-pS672, a marker of Cyclin D-CDK4 activity (Figure 1D) (13).

To measure the impact of RB and p130 loss on cell cycle gene expression after DNA damage, we performed RNA-seq of sgP130, sgRB1, sgRB1+sgP130 and Control cells treated with doxorubicin. We performed differential gene expression analysis between doxorubicin treated samples to generate a heat-map of the top 250 significantly altered genes (Figure 1E). Many (75) of the top 250 differentially expressed genes were G1/S genes while fewer (17) G2/M genes were differentially expressed (Figure 1E). Control and sgP130 cells had similarly lower levels of differentially expressed genes compared to sgRB1 and sgRB1+sgP130 cells. Strikingly, sgRB1+sgP130 cells showed a stronger differential effect than the sgRB1 cells. A volcano plot comparing Log_2_ fold change and adjusted *P* value revealed that the most significantly up-regulated genes were predominantly G1/S genes including *MCM5*, *ASF1B, CDT1*, *E2F1* and *FAM111B* (Figure 1F). In contrast, the down-regulated genes were not predominately cell cycle regulated. Furthermore, very few direct p53 target genes were differentially expressed, consistent with the observation that they were activated to similar levels in response to doxorubicin in each of the cell lines when compared by pairwise analysis and by RT-qPCR of p21 (*CDKN1A*) and PUMA (Supplementary Figure S1A–C). Changes in expression between cell lines did not reflect differences in basal levels of expression as untreated cells had very few differentially expressed genes (Supplementary Figure S1D and E).

### p53 and p21 are required to repress G1/S and G2/M genes after DNA damage

**Figure 2.**
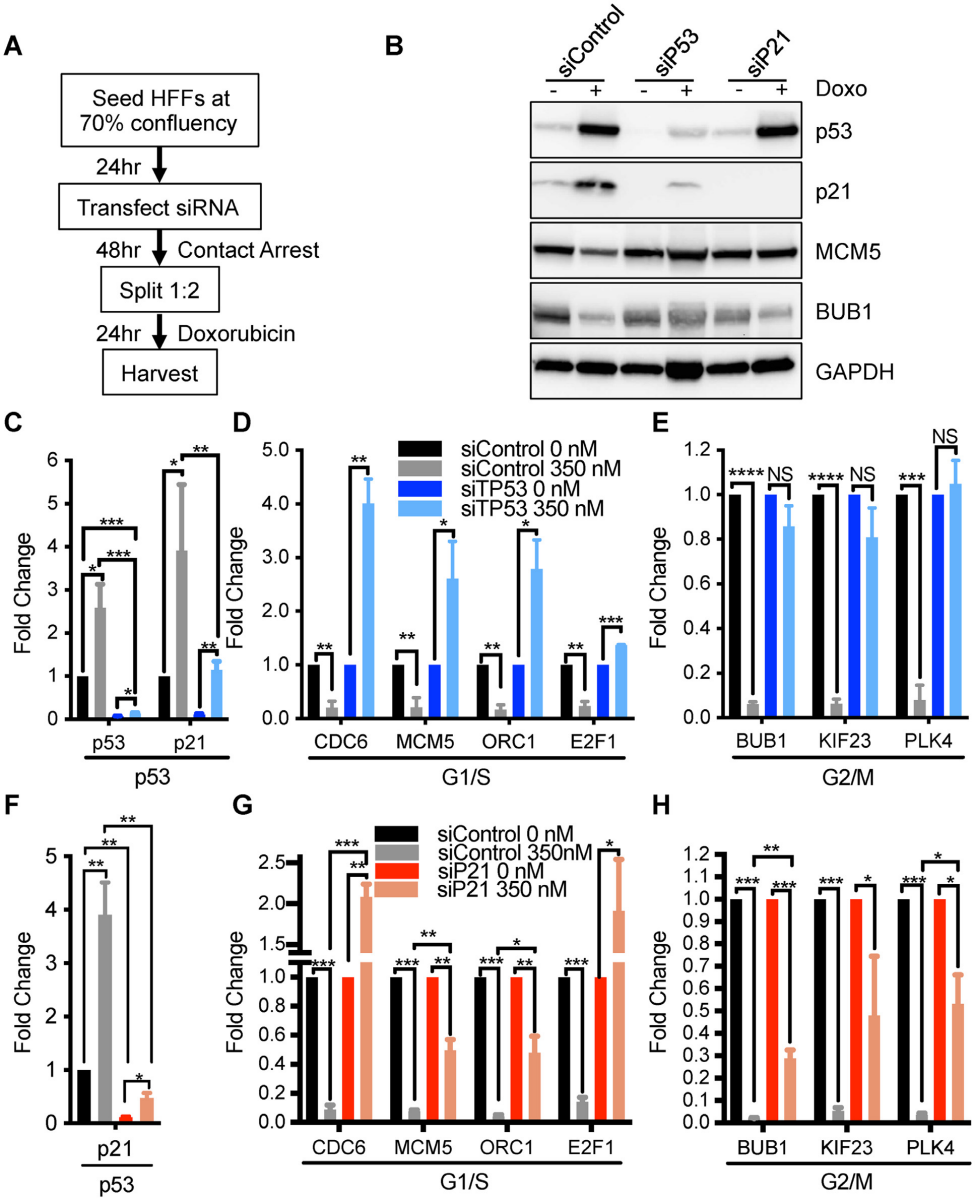
Reduced levels of cell cycle genes after DNA damage is dependent on p53 and p21 in HFFs. (**A**) Schematic of experimental design for B–H. (**B**) Parental HFFs transfected with indicated siRNA were released from contact arrest into 350 nM doxorubicin and assessed by immunoblot after 24 h. (**C**) Same as B, but cells transfected with siTP53 were assessed by RT-qPCR for p53 target genes normalized to siControl untreated cells (n = 3). (**D** and **E**) Same as C but fold change for G1/S (**D**) or G2/M (**E**) genes was normalized to untreated for each siRNA condition (n = 3). (**F**) Same as B, but cells transfected with siP21 were assessed by RT-qPCR for p53 target genes normalized to siControl untreated cells (*n* = 3). (D and E) Same as F but fold change for G1/S (**G**) or G2/M (**H**) genes was normalized to untreated for each siRNA condition (n = 3). Student's t-test was used to measure significance between indicated samples. *P* values are as indicated as * for <0.05, ** for <0.01, *** for <0.001, and **** for <0.0001. NS indicates *P* value >0.05.

To determine the contribution of p53 and p21 to repression of G1/S and G2/M genes, HFFs were transfected with siRNA against *TP53*, *CDKN1A*, or a control sequence for 48 h, then split into fresh media containing doxorubicin and harvested 24 h later (Figure 2A). Doxorubicin treatment of siControl cells led to increased levels of p53 and p21 protein and mRNA (Figure 2B and C), and a significant reduction in levels of several G1/S genes (*CDC6*, *E2F1*, *MCM5* and *ORC1*) and G2/M genes (*BUB1*, *KIF23*, and *PLK4*) (Figure 2D and E). In contrast, when p53 was knocked down, we observed significantly increased levels of G1/S genes and loss of repression of G2/M genes in response to doxorubicin. Similarly, in cells with p21 knockdown, doxorubicin treatment led to increased levels of the G1/S genes *CDC6* and *E2F1* compared to control siRNA cells (Figure 2F and G). Levels of *MCM5* and *ORC1* were also significantly higher in the p21 knockdown cells compared to the control cells in response to doxorubicin. Levels of several G2/M genes were also significantly de-repressed in the p21 knockdown cells compared to control knockdown cells in response to doxorubicin (Figure 2H). These data indicate that repression of G1/S and G2/M genes in HFFs after DNA damage was dependent on p53 and, at least in part, on p21.

### RB is required to repress G1/S genes after p53 activation

**Figure 3.**
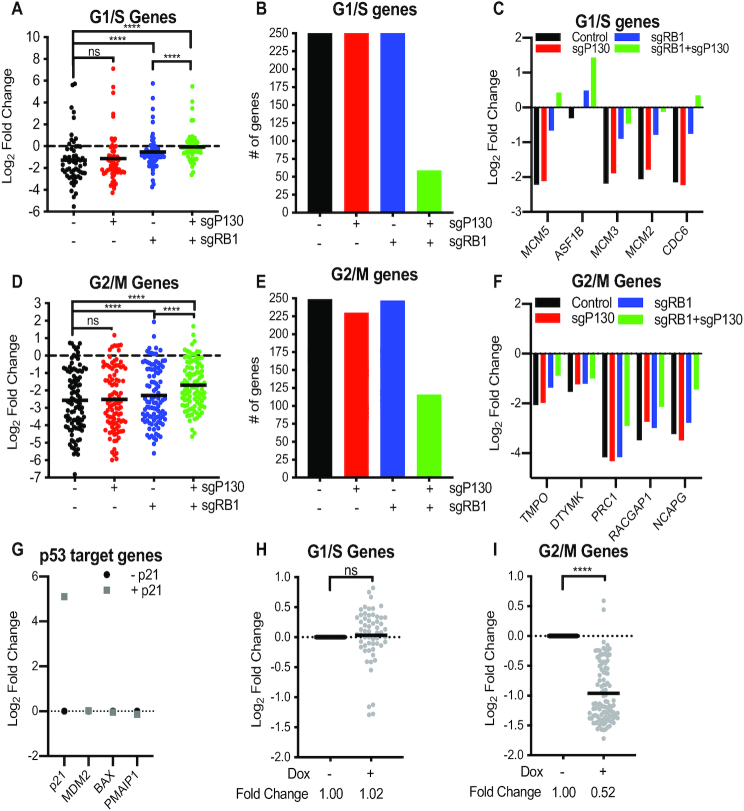
G1/S and G2/M genes are differentially repressed after p53 activation when RB or p130 are lost. (**A**) Log_2_ fold change of high stringency G1/S genes measured by differential gene expression analysis comparing 0–350 nM doxorubicin treated samples for each cell line (*n* = 2). Statistical analysis performed using Wilcoxon matched-pairs signed rank test. (**B**) Number of G1/S genes differentially expressed in pairwise differential gene expression analysis. (**C**) Log_2_ fold change of G1/S genes in pairwise analysis from 3A. Gene list generated from genes with lowest adjusted *P* value in analysis from 1E. (**D**) Log_2_ fold change of high stringency G2/M genes measured by differential gene expression analysis comparing 0–350 nM doxorubicin treated samples for each cell line (*n* = 2). Statistical analysis performed using Wilcoxon matched-pairs signed rank test. (**E**) Number of G2/M genes differentially expressed in pairwise differential gene expression analysis. (**F**) Log_2_ fold change of G2/M genes in pairwise analysis from 3D. Gene list generated from genes with lowest adjusted *P* value in analysis from 1E. (**G**) RNA-seq of SaOS-2 cells with doxycycline inducible p21 expression (*n* = 3). Log_2_ fold change of p53 target genes. (H and I) Differential gene expression levels of high stringency G1/S (**H**) and G2/M (**I**) genes after p21 induction in SaOS-2 cells. Statistical test with Wilcoxon matched-pairs signed rank test. *P* values indicated as *<0.05, **<0.01, ***<0.001, ****<0.0001, NS: non significant. See also Supplemental Figures S2–S4.

Given the distinct effects of RB and p130 loss on G1/S and G2/M genes in response to doxorubicin, we suspected that G1/S and G2/M genes were differentially repressed by RB and DREAM complexes. First, we asked whether loss of RB or p130 affects expression of a list of high-stringency G1/S genes (61 genes) (Figure 3A). We performed pairwise analysis of each cell line in the presence or absence of doxorubicin and measured the Log_2_ fold change of these genes. Loss of RB alone significantly reduced the level of repression on G1/S genes by doxorubicin and the combined loss of RB and p130 nearly eliminated the repression effect (Figure 3A). Similar to the effect on the high stringency G1/S genes response, levels of a list of lower stringency G1/S genes (573 genes) were also affected by doxorubicin treatment (Figure 3B). More than 300 G1/S genes were differentially expressed in the Control, sgP130, and sgRB1 knockout cells, while only 59 of 573 G1/S genes changed significantly in sgRB1+sgP130 cells. We identified the top 5 most differentially expressed genes from Figure 1E and plotted the Log_2_ fold change for *MCM5*, *ASF1B*, *MCM3*, *MCM2* and *CDC6* (Figure 3C). Combined loss of RB and p130 resulted in the largest increase in G1/S gene expression compared to sgRB1 cells, demonstrating cooperative repression by p130 and RB. While loss of p130 alone did not strongly change the downregulation of these genes, and loss of RB led to a partial loss of repression, loss of both RB and p130 had near complete loss of repression of G1/S genes. To validate these observations, we performed RT-qPCR for several G1/S genes and found a similar effect (Supplementary Figure S2A-D). These results indicate that the double knockout sgRB1+sgP130 cells had impaired control of G1/S genes after DNA damage compared to that observed in cells with loss of RB or p130 alone.

Next, we tested if doxorubicin induced repression of cell cycle genes was generalizable to other methods of p53 activation by using RG7388, a potent MDM2 inhibitor (Supplementary Figure S3) (43). Parental HFFs were split into RG7388 and assessed for p53 response after 24 h (Supplementary Figure S3A). We observed an increase in p53 and p21 protein levels in response to RG7388. MDM2 inhibition led to similar levels of p53 activation regardless of RB or p130 status as measured by the increase in p21 mRNA and p53-pS15 protein levels (Supplementary Figure S3B-C). Similar to the doxorubicin treated cells, MDM2 inhibitor treated control and p130 knockout cells showed a significant reduction in the levels of the G1/S genes *MCM5* and *E2F1* (Supplementary Figure S3B, S3D-E). In contrast, cells lacking RB alone (sgRB1) or both RB and p130 (sgRB1+sgP130) had a decreased response of *MCM5* and *E2F1* to MDM2 inhibition. Differences in gene expression were not due to differences in cell cycle status (Supplementary Figure S3F). These data indicate that RB was required for reduced levels of G1/S genes after p53 activation by either doxorubicin or MDM2 inhibition.

### P130 contributes to the repression of G2/M gene expression after p53 activation

Since p130 binds to G2/M gene promoters by CHR elements and RB is not known to significantly occupy G2/M promoters, we suspected that p130 could contribute to repression of G2/M promoters after p53 activation. To determine the effect of p130 and RB loss on G2/M genes after doxorubicin treatment, the pairwise Log_2_ fold change of a list of high stringency G2/M genes (104 genes in each cell line) was assessed (Figure 3D). Levels of G2/M genes were strongly repressed in all four cell lines (Figure 3D). Loss of RB and p130 in combination resulted in a partial rescue of expression of high stringency G2/M genes while sgRB1+sgP130 cells was significantly higher (Figure 3D). Notably, more than 225 genes from a list of 433 low stringency G2/M genes were differentially expressed after doxorubicin treatment of Control, sgP130, and sgRB1 cells. In contrast, less than half of these genes were differentially expressed in the sgRB1+sgP130 cells (Figure 3E). While we observed a partial rescue of G2/M genes in sgRB1+sgP130 cells, levels were not restored to baseline after doxorubicin treatment (Figure 3F, Supplemental Figure S2E).

To determine if loss of RB resulted in deregulation of G1/S and G2/M genes in a cancer cell line, we employed SaOS-2 osteosarcoma cells. SaOS-2 cells are null for RB and p53 but can undergo cell cycle arrest after exogenous expression of p53 or p21 (44,45). In addition, SaOS-2 cells can form an intact DREAM complex in response to exogenous expression of RB (46). We performed RNA-seq with SaOS-2 cells engineered with a doxycycline-inducible p21 vector (44). RNA was collected from SaOS-2 cells with and without doxycycline induced p21 expression (Figure 3G-I, Supplemental Figure S4). As expected, induction of p21 led to increased levels of *p21* transcript but no increases in other p53 response genes including *MDM2*, *BAX* or *PMAIP1* (Figure 3G). Similar to HFFs lacking RB, SaOS-2 cells were unable to repress G1/S genes after p21 induction, consistent with a requirement for RB to repress G1/S genes (Figure 2H). In contrast, G2/M genes were significantly repressed upon p21 induction, indicating that RB was not required for repression of G2/M genes (Figure 3I). Consistent with our observations with HFFs, the absence of RB nearly eliminated repression of G1/S genes but had a limited impact on repression of G2/M genes.

### P107 represses G1/S and G2/M gene expression

**Figure 4.**
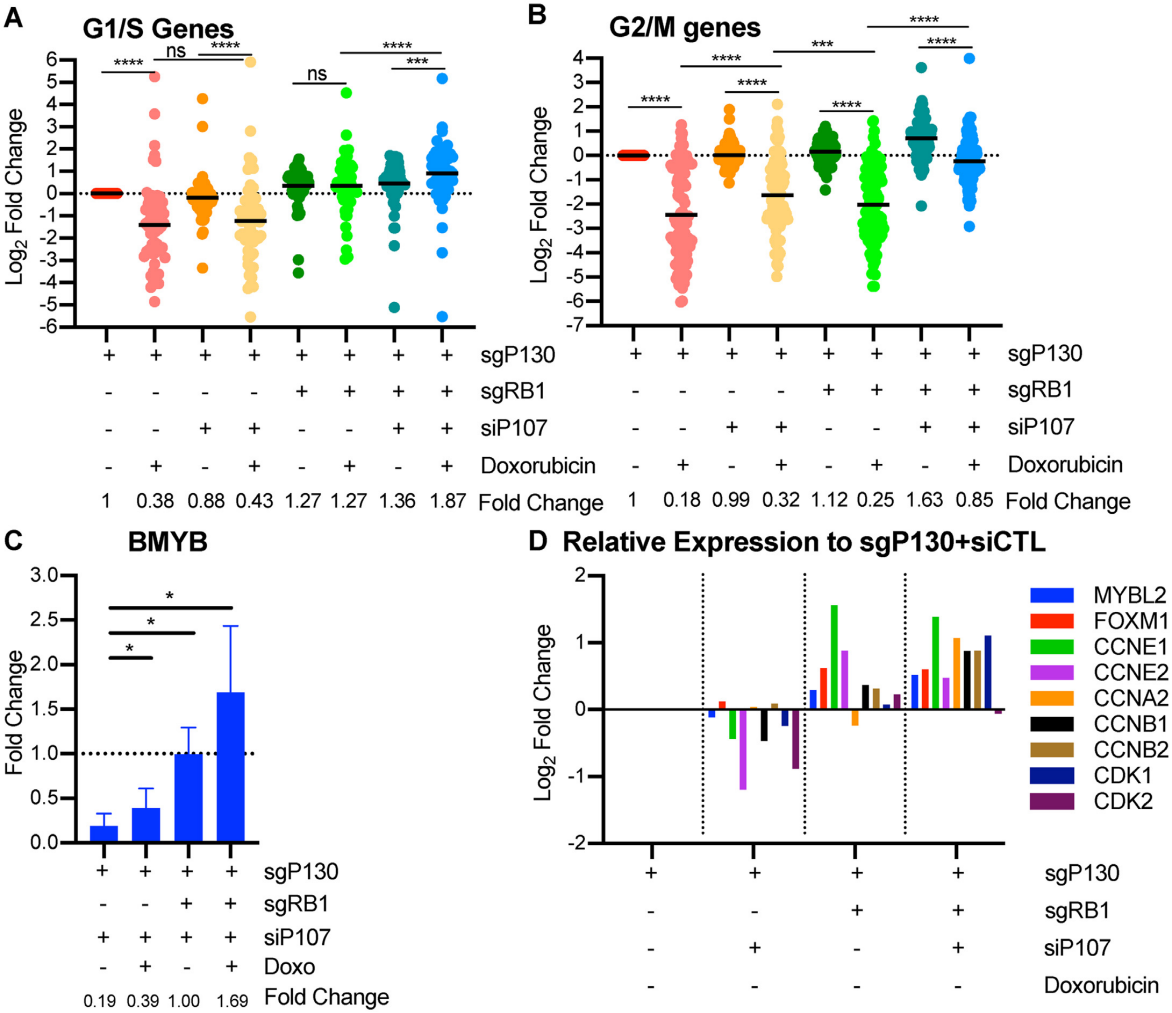
p107 contributes to repression of G1/S and G2/M gene expression after p53 activation in absence of p130 or RB. (**A**) sgP130 or sgRB1+sgP130 HFFs were seeded at 70% density, transfected 24 h later with siRNA against p107 or Control sequence, and split from contact arrest after 48 h into doxorubicin (350 nM). Cells were collected 24 h after release and assessed by RNA-seq and differential gene expression analysis was completed compared to sgP130+siCTL untreated. Expression of high stringency G1/S genes was measured. Statistical analysis using Wilcoxon matched-pairs signed rank test between indicated samples (*n* = 2). (**B**) Same as (A) but with high stringency G2/M genes. (**C**) Same as (A) but transcript levels of BMYB were measured by RT-qPCR and fold change was calculated in relation to untreated for each cell type. Statistical analysis using Student's T-test between indicated samples (*n* = 3). (**D**) Relative expression of MMB-FOXM1 complex and associated genes measured compared to sgP130+siCTL untreated cells using RNA-seq. *P* values indicated as *<0.05, **<0.01, ***<0.001, ****<0.0001, NS: non significant. See also Supplementary Figures S5 and S6.

Given the increase in p107 protein levels in p130 knockout cells (Figure 1A) and its ability to bind to E2F4 and MuvB, we tested if p107 could contribute to repression of the G2/M genes in response to p53 activation. SgP130 and sgRB1+sgP130 cells were transfected with siRNA against p107 or a control sequence, released from contact arrest by splitting and replating followed by doxorubicin treatment for 24 h. Knockdown of p107 in both cell lines led to a significant decrease in the p107 mRNA transcript as measured by RT-qPCR and normalized mean counts (Supplementary Figure S5A and B). Levels of p53 direct target genes were significantly increased in response to doxorubicin with a mean fold change of approximately 3 fold in both cell lines regardless of p130, RB, or p107 status (Supplementary Figure S5C). We performed pairwise differential gene expression analysis of each condition compared to untreated sgP130+siControl cells. As expected, doxorubicin treatment led to reduced expression of high stringency G1/S genes in sgP130 cells, but the added knockdown of p107 led to a modest de-repression from 0.38- to 0.43-fold (Figure 4A, Supplemental Figure S5D). Consistent with earlier results, doxorubicin had a reduced effect on repression of G1/S gene in sgRB1+sgP130 cells, while knockdown of p107 led to a further de-repression of G1/S genes, increasing levels from 1.27 to 1.87 fold.

Next, we measured the effect of p107 loss on expression of G2/M genes in cells treated with doxorubicin (Figure 4B, Supplemental Figure S5E). Remarkably, loss of p107 in the sgP130 single knockout cells led to a significant de-repression of high-stringency G2/M genes. The effect was even more pronounced in sgRB1+sgP130 cells, a finding validated by RT-qPCR for *PLK4* and *BUB1* (Supplementary Figure S5F and G). We confirmed the phenotype using two independent siRNAs or pooled siRNA against p107 in sgRB1+sgP130 cells and found significant de-repression of several high stringency G2/M genes including *BUB1*, *KIF23*, and *PLK4* (Supplementary Figure S6). These findings indicate that p107 contributes to the repression of G1/S and G2/M genes after DNA damage when p130 or RB are lost. Importantly, G2/M gene expression was significantly higher in doxorubicin treated cells lacking p130 and p107 compared to those lacking RB and p130, indicating that loss of p107 and p130 de-repressed G2/M genes to a greater degree than loss of RB and p130 (Figure 4B).

Since RB does not directly bind the promoters of G2/M genes that lack E2F sites, its repression of G2/M genes is likely to be indirect. One possible role for RB in the repression of G2/M genes could be regulation of the levels of the MMB-FOXM1 components, including several that are G1/S genes (13). To address this model, we assessed the expression of BMYB (*MYBL2*) after doxorubicin treatment in sgP130 or sgRB1+sgP130 cells with siP107 (Figure 4C). We found loss of RB, p130, and p107 significantly increased the expression of BMYB with cells lacking all three having the highest expression of BMYB. Furthermore, we found that even in untreated samples, sgRB1+sgP130 cells or sgRB1+sgP130+siP107 cells had significant increased levels of MMB-FOXM1 complex components as well as Cyclin-CDKs that are known positive regulators of this complex (Figure 4D). Previous reports demonstrate that B-MYB and FOXM1 promoter recruitment follows their mRNA and protein levels during the cell cycle (19,20). These data indicate that RB control of G1/S genes that encode for BMYB and Cyclin-CDKs correlates with increased expression of G2/M genes and may explain its indirect control of G2/M gene expression.

### P107 repression of G2/M genes controls entry into mitosis

**Figure 5.**
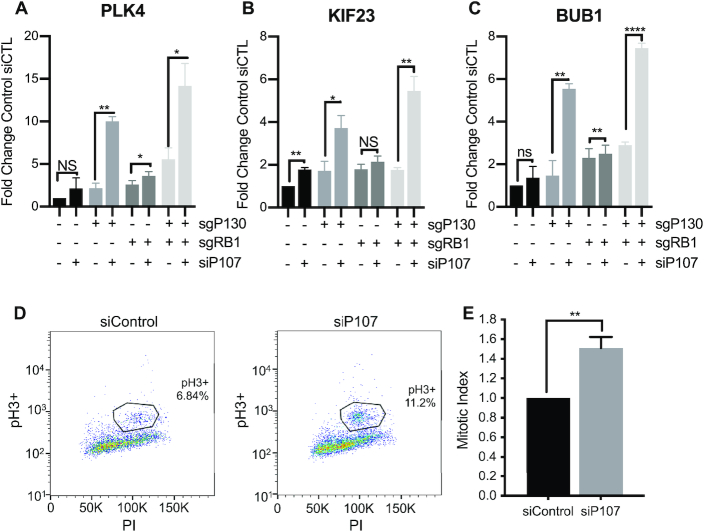
P107 repression of G2/M genes prevents mitotic entry. (**A–C**) RT-qPCR of HFFs with indicated genotype transfected with siRNA and harvested after 48 h of contact arrest and serum starvation (*n* = 3). (**D**) sgRB1+sgP130 cells were transfected with siRNA, contact arrested for 48 h, and then were split into nocodazole and harvested at 30 h and analyzed by flow cytometry using antibody against phospho-H3 and propidium iodide. (**E**) Quantification of pH3+ cells in D. Student's t-test was used to measure significance between indicated samples (*n* = 3). *P* values are as indicated as * for <0.05, ** for <0.01 and *** for <0.001. NS indicates *P* value >0.05.

Given the contribution of p107 to repressing G2/M genes under DNA damage conditions, we asked if p107 could contribute to repression of G2/M genes *PLK4, KIF23*, and *BUB1* in quiescent cells (Figure 5A–C). Using a previously reported method of inducing quiescence in HFF cells lacking RB and p130, cells were seeded at 70% confluency, transfected with siRNA after 24 h, and then serum starved and contact arrested for an additional 48 h (13). Knockdown of p107 in cells lacking p130 alone or lacking both p130 and RB led to a significant increase in levels of several G2/M genes in quiescent G0 cells. Since the impact of p107 loss on repression of G2/M genes in Control and sgRB1 cells was quite modest, p130 likely plays a dominant role to p107 when it is present (Figure 5A–C).

Given the ability of p107 to repress G2/M genes, we tested if p107 contributed to the entry of cells into mitosis. sgRB1+sgP130 cells were transfected with siControl or siP107, treated with nocodazole to capture cells that have entered mitosis, and analyzed by flow cytometry (Figure 5D). Knockdown of p107 led to a significant increase in the mitotic index (Figure 5E). This data provides a specific and functional role for p107 as a repressor of the G2/M genes during quiescence, DNA damage, and progression to mitosis.

## DISCUSSION

Here, we demonstrate that G1/S and G2/M genes are differentially regulated by RB, p130 and p107 in response to DNA damage. We have previously reported that RB and p130 cooperate to repress G1/S gene expression during quiescence (13). Here, we observed a similar effect where RB and p130 cooperate to repress G1/S genes after p53 activation. RB has a dominant role in reducing levels of the early G1/S genes in quiescence and in response to p53 activation while loss of p130 alone does not affect G1/S gene expression. We found an additional role for p107 in repression of G1/S genes after p53 activation in cells lacking RB and p130. In contrast to the effect on G1/S genes, G2/M genes were specifically repressed by p130 and p107 in response to doxorubicin even in the presence of RB. G2/M genes were de-repressed after p107 knockdown in sgP130 cells as well as in sgRB1+sgP130 cells in quiescent conditions and in response to p53 activation. Furthermore, we observed that p107 repression of G2/M genes restricts entry into mitosis.

We provide evidence for a specific and previously unappreciated role for p107 as a repressor of G2/M gene expression during p53 activation, quiescence, and proliferation. Normally, p107 is not expressed during G0 and early G1 when DREAM is active. Since p107 is a G1/S gene, its peak expression occurs during S phase when p130 is inactivated by cyclin-dependent kinase phosphorylation and degradation (25,47). In sgP130 cells, p107 levels are increased and contribute to specific repression of G2/M genes in quiescence conditions and in response to doxorubicin. Chromatin occupancy studies of endogenous p107 in non-transformed cells have been technically challenging. However, p107 directly binds MuvB or E2F4 in cells lacking p130 and therefore is likely to bind to promoters of G1/S and G2/M genes containing E2F4 and MuvB binding sites. Although p107 has been shown to interact with MuvB in cells and *in vitro*, no specific role for p107 in cell cycle gene repression has been previously reported (12,13,22,24). p130 and p107 compensate for each other during mouse development, as double knockout mice displayed severe developmental defects and delays while single knockouts of either p130 or p107 were viable and fertile (27,47–49).

Although we found a specific role for p130, p107 and RB in repression of G2/M genes in response to doxorubicin, the levels of many G2/M genes remain at least partially reduced after doxorubicin treatment (Figure 4B). This persistent effect may reflect reduced activation of the MMB-FOXM1 complex by p21 mediated inhibition of CDK activity. Cyclin A-CDK phosphorylation of BMYB and FOXM1 contributes to the ability of MMB-FOXM1 to *trans*-activate G2/M genes (50–52). BMYB binding to MuvB facilitates interaction with CHR elements in the G2/M gene promoters (17,33,53). It has also been reported that BMYB has reduced binding to the MuvB core under DNA damage conditions. These effects of p53 and p21 on CDK activity and the MMB-FOXM1 complex may explain why loss of RB, p130, and p107 did not completely restore G2/M gene levels in doxorubicin treated cells (Figure 4B).

The use of primary HFFs enabled specific interrogation of the effects of the three pocket proteins (RB, p130 and p107) in repression of G1/S and G2/M genes after p53 activation. By specifically reducing levels of each pocket protein, we were able to test the individual contributions of RB, p130, and p107 without affecting the MuvB complex, a key activator of G2/M gene expression as part of the MMB-FOXM1 complex. Repression of G2/M genes and inhibition of entry into mitosis by the combined activity of p130 and p107 may be relevant in cancer cells that have disrupted G1/S checkpoint. Cells containing a *RB1* mutation, overexpression of Cyclin D, or loss of p16 have reduced ability to repress G1/S genes in response to p53 and p21 activation. However, as shown here, p130 and p107 cooperate to repress the G2/M genes and reduce entry into mitosis in response to p53 activation even in the absence of RB. Given these effects, cancer cells with mutated RB and p53 would be expected to have reduced ability to repress both G1/S and G2/M genes. Consistent with this model, we demonstrated that exogenous expression of p21 in SaOS-2 cells led to significant repression of G2/M genes.

A recent study examined the control of gene expression after p53 activation by knocking out LIN37, a component of the MuvB core (54). Knockout of LIN37 could affect the ability of p130 to form the DREAM complex, p107 to bind MuvB, and assembly of the MMB-FOXM1 complex leading to indeterminate effects on expression of G1/S and G2/M genes. The presence of a variety of oncogenic mutations in cancer cell lines may also indirectly affect the dependencies on RB, p130, and p107. The use of primary, non-immortalized, early passage fibroblasts enabled the study of specific roles of RB, p130 and p107 without confounding influences of oncogenes or perturbations in the MMB-FOXM1 complex (1,55,56). Nevertheless, investigation of RB, p130, and p107 control of cell cycle gene expression in the context of other oncogenic mutations could provide significant insight into how cell cycle progression is regulated in cancer cells treated with DNA damaging chemotherapeutics. Furthermore, analysis of BMYB and FOXM1 activity in cancer cells lacking RB and p130 could provide significant insight into the activation of G2/M gene expression when G1/S gene expression is deregulated.

**Figure 6.**
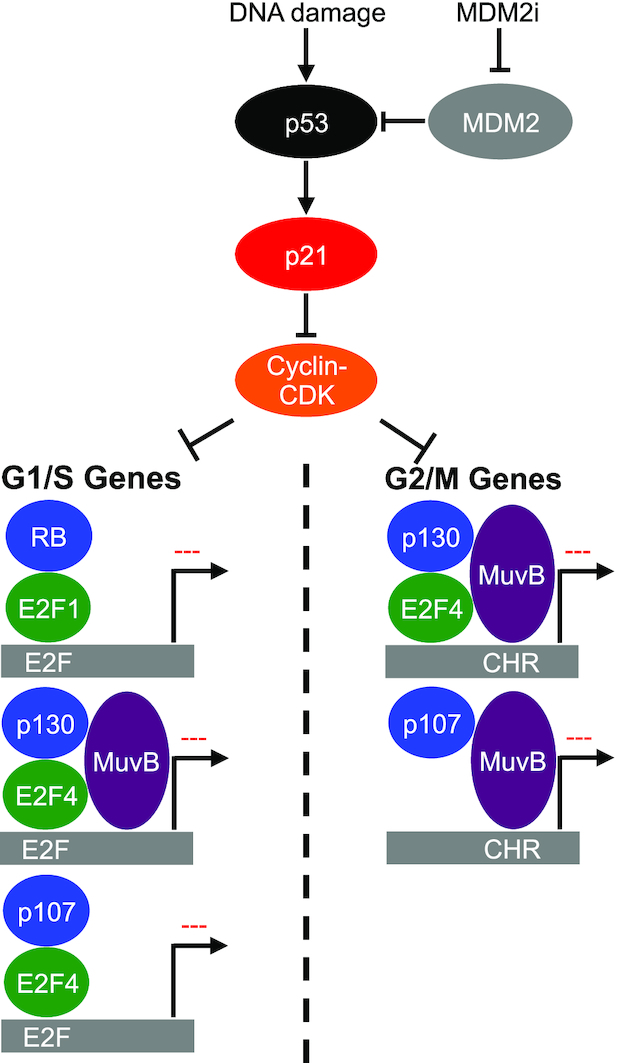
Model for DREAM and RB repression of cell cycle gene expression after p53 activation. P53 activation by DNA damage or inhibition of MDM2 results in increased expression of p21 and inhibition of Cyclin E-CDK2. Decreased CDK2 activity results in restoration of RB and DREAM mediated repression of G1/S gene expression. Under these conditions, DREAM and p107-DREAM are able to bind and repress G1/S and G2/M genes, promoting cell cycle arrest.

In conclusion, we demonstrate p53 mediated down-regulation of cell cycle genes can be functionally separated into the repression of G1/S genes by RB, with contributing effects of p130 and p107, and repression of G2/M genes by p130 and p107 (Figure 6). Together, our results support a model of control of cell cycle gene expression by p53 where RB, p130 and p107 cooperate to repress G1/S genes while p130 and p107 repress G2/M genes.

## DATA AVAILABILITY

Sequence data are deposited at GEO: GSE128669; GSE128711; GSE135842.

## Supplementary Material

gkz961_Supplemental_Files
