# Supplementary material for: RB, p130 and p107 differentially repress G1/S and G2/M genes after p53 activation
Source: Nucleic Acids Res. 2019 Oct 31;47(21):11197–208. doi: 10.1093/nar/gkz961 (PMC6868438; doi:10.1093/nar/gkz961)
Supplement: gkz961_Supplemental_Files [file gkz961_supplemental_files.zip › Schade et al Supplemental Figures.pptx]

## Slide 1
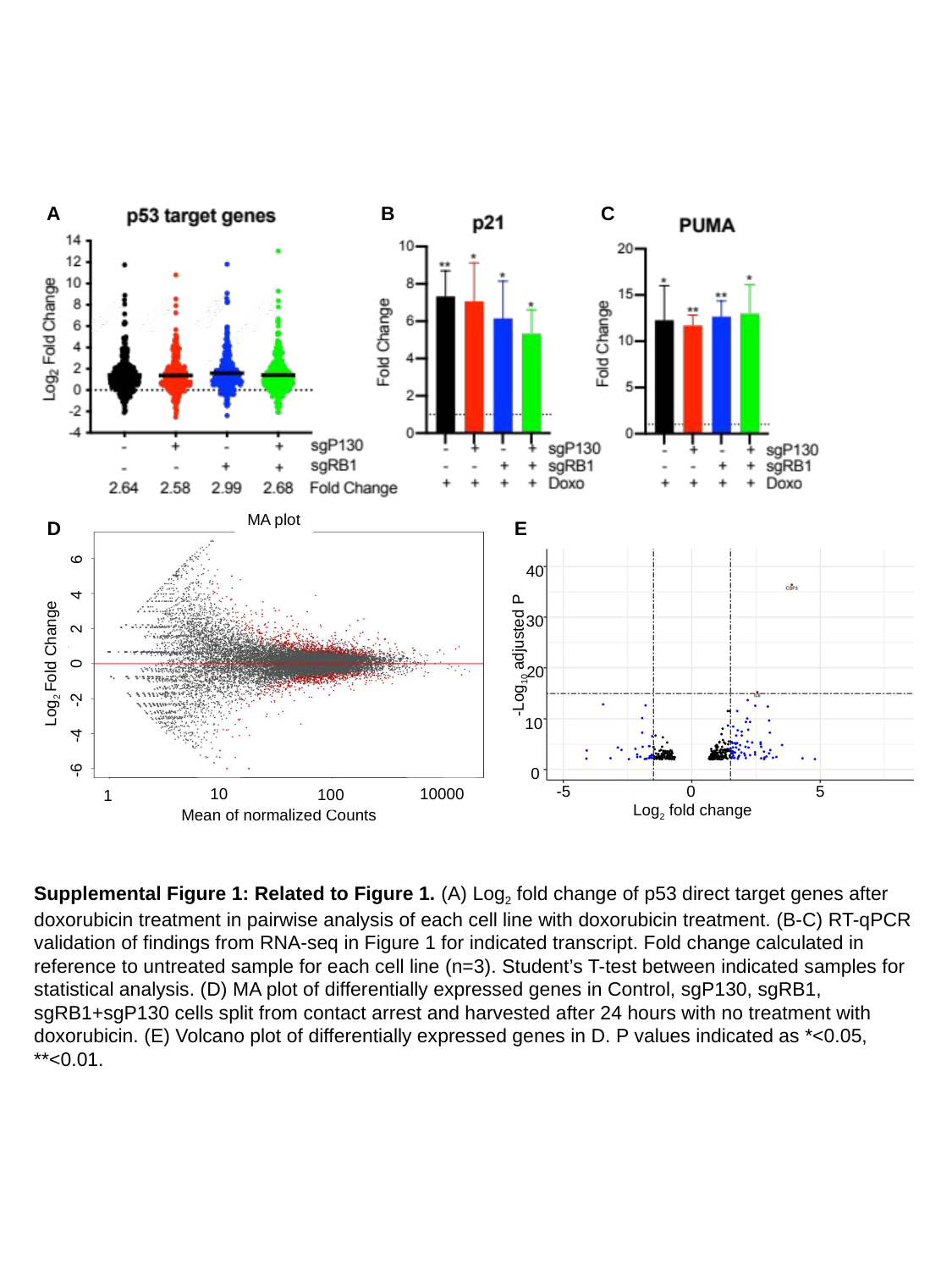

A
B
C
MA plot
6
4
2
Log2 Fold Change
0
-2
-4
-6
10
10000
100
1
Mean of normalized Counts
D
E
40
30
-Log10 adjusted P
20
10
0
-5
0
5
Log2 fold change
Supplemental Figure 1: Related to Figure 1. (A) Log2 fold change of p53 direct target genes after doxorubicin treatment in pairwise analysis of each cell line with doxorubicin treatment. (B-C) RT-qPCR validation of findings from RNA-seq in Figure 1 for indicated transcript. Fold change calculated in reference to untreated sample for each cell line (n=3). Student’s T-test between indicated samples for statistical analysis. (D) MA plot of differentially expressed genes in Control, sgP130, sgRB1, sgRB1+sgP130 cells split from contact arrest and harvested after 24 hours with no treatment with doxorubicin. (E) Volcano plot of differentially expressed genes in D. P values indicated as *<0.05, **<0.01.

## Slide 2
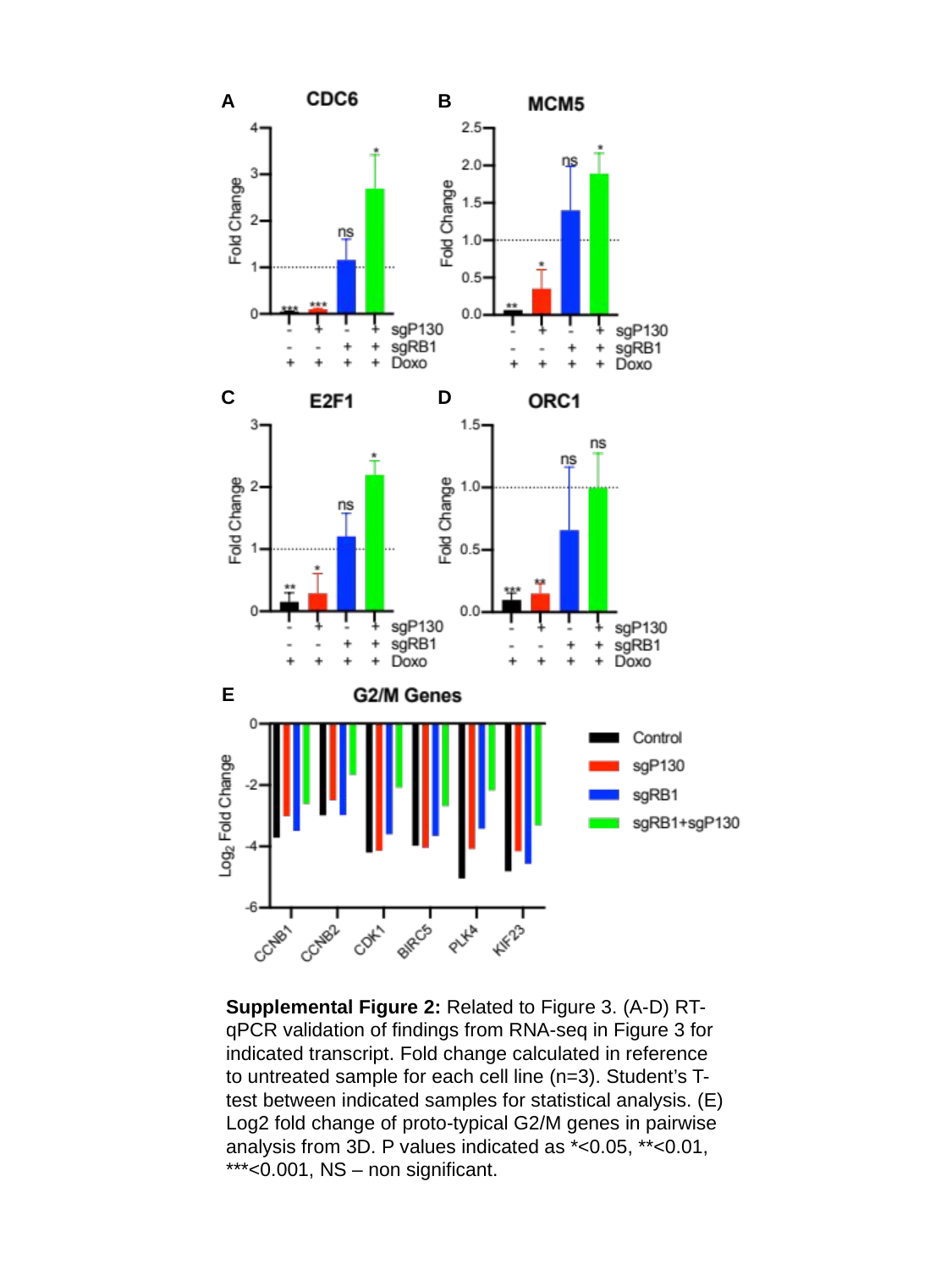

A
B
C
D
E
Supplemental Figure 2: Related to Figure 3. (A-D) RT-qPCR validation of findings from RNA-seq in Figure 3 for indicated transcript. Fold change calculated in reference to untreated sample for each cell line (n=3). Student’s T-test between indicated samples for statistical analysis. (E) Log2 fold change of proto-typical G2/M genes in pairwise analysis from 3D. P values indicated as *<0.05, **<0.01, ***<0.001, NS – non significant.

## Slide 3
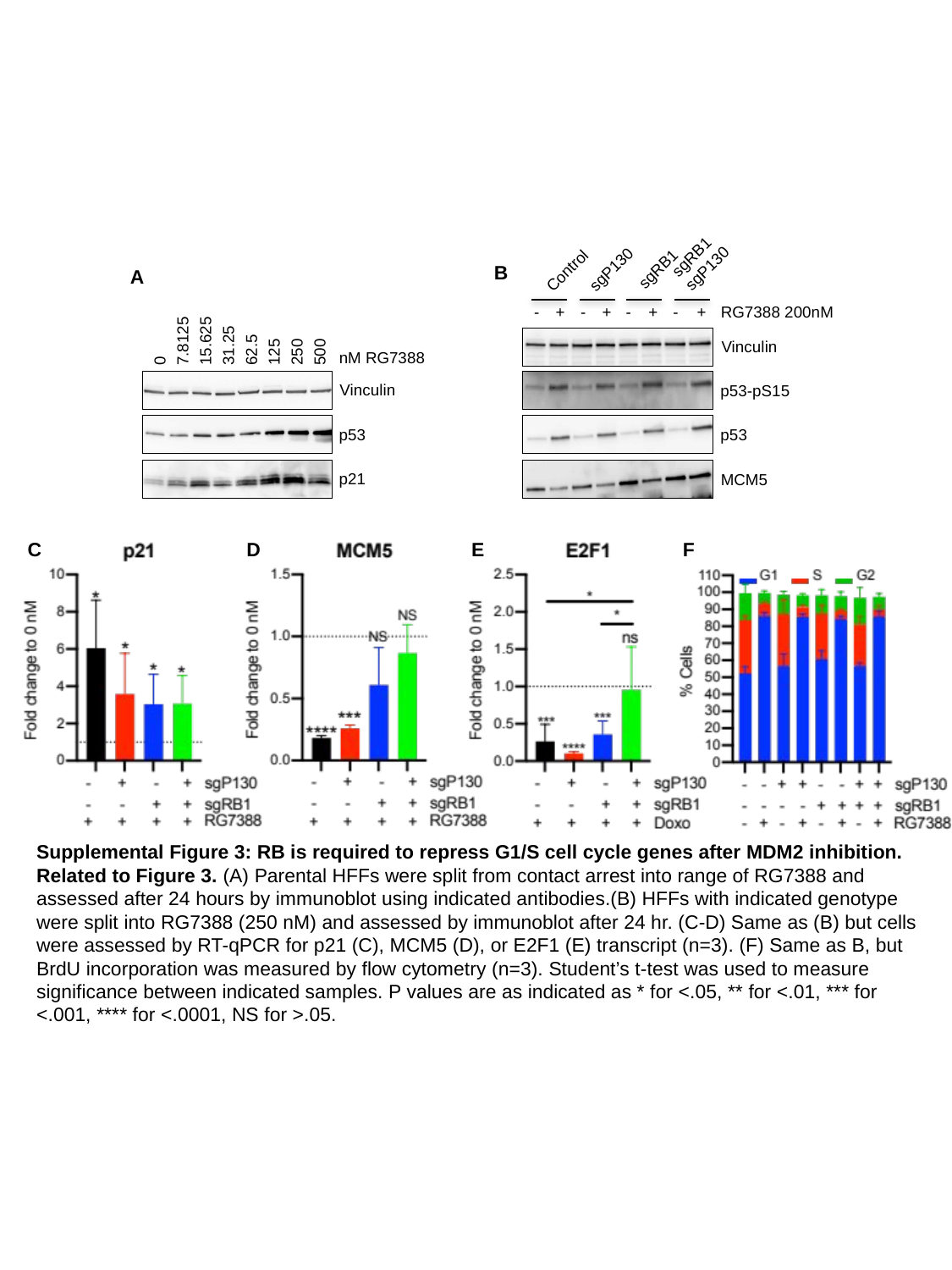

sgRB1
sgP130
sgRB1
sgP130
Control
-
+
-
+
-
+
-
+
RG7388 200nM
Vinculin
p53-pS15
p53
MCM5
B
A
0
7.8125
15.625
31.25
62.5
125
250
500
nM RG7388
Vinculin
p53
p21
C
D
E
F
Supplemental Figure 3: RB is required to repress G1/S cell cycle genes after MDM2 inhibition. Related to Figure 3. (A) Parental HFFs were split from contact arrest into range of RG7388 and assessed after 24 hours by immunoblot using indicated antibodies.(B) HFFs with indicated genotype were split into RG7388 (250 nM) and assessed by immunoblot after 24 hr. (C-D) Same as (B) but cells were assessed by RT-qPCR for p21 (C), MCM5 (D), or E2F1 (E) transcript (n=3). (F) Same as B, but BrdU incorporation was measured by flow cytometry (n=3). Student’s t-test was used to measure significance between indicated samples. P values are as indicated as * for <.05, ** for <.01, *** for <.001, **** for <.0001, NS for >.05.

## Slide 4
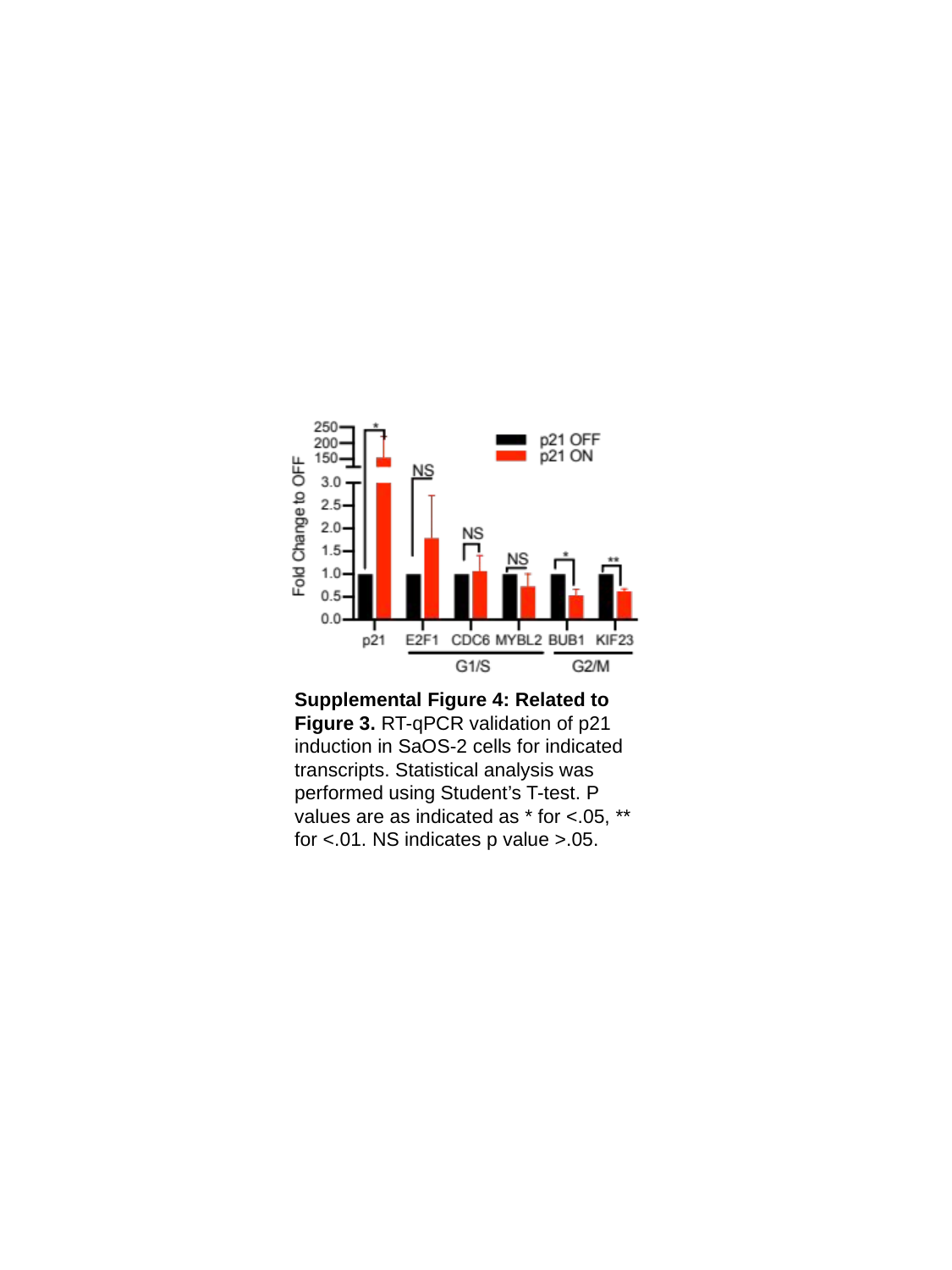

Supplemental Figure 4: Related to Figure 3. RT-qPCR validation of p21 induction in SaOS-2 cells for indicated transcripts. Statistical analysis was performed using Student’s T-test. P values are as indicated as * for <.05, ** for <.01. NS indicates p value >.05.

## Slide 5
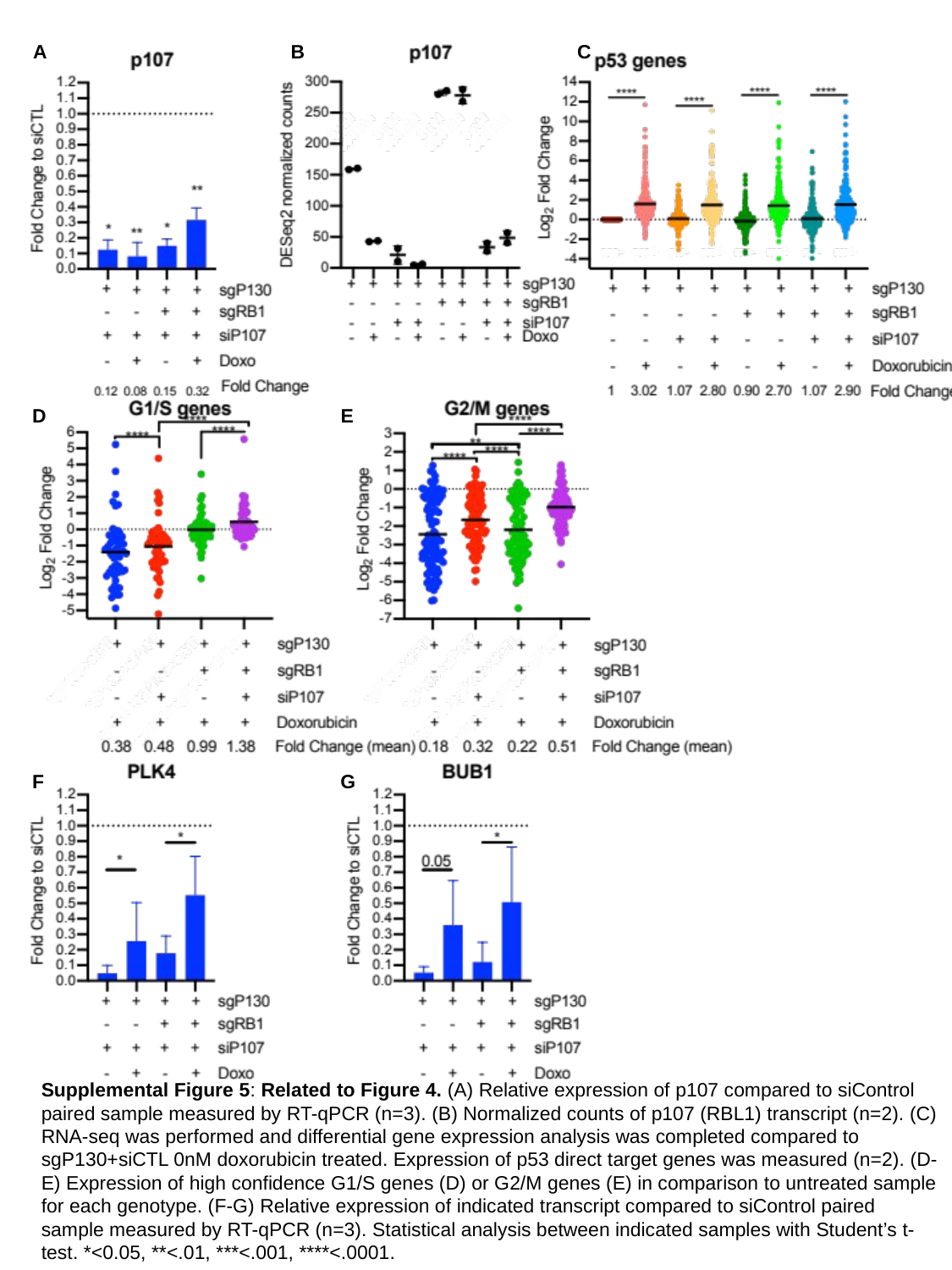

A
B
C
D
E
F
G
Supplemental Figure 5: Related to Figure 4. (A) Relative expression of p107 compared to siControl paired sample measured by RT-qPCR (n=3). (B) Normalized counts of p107 (RBL1) transcript (n=2). (C) RNA-seq was performed and differential gene expression analysis was completed compared to sgP130+siCTL 0nM doxorubicin treated. Expression of p53 direct target genes was measured (n=2). (D-E) Expression of high confidence G1/S genes (D) or G2/M genes (E) in comparison to untreated sample for each genotype. (F-G) Relative expression of indicated transcript compared to siControl paired sample measured by RT-qPCR (n=3). Statistical analysis between indicated samples with Student’s t-test. *<0.05, **<.01, ***<.001, ****<.0001.

## Slide 6
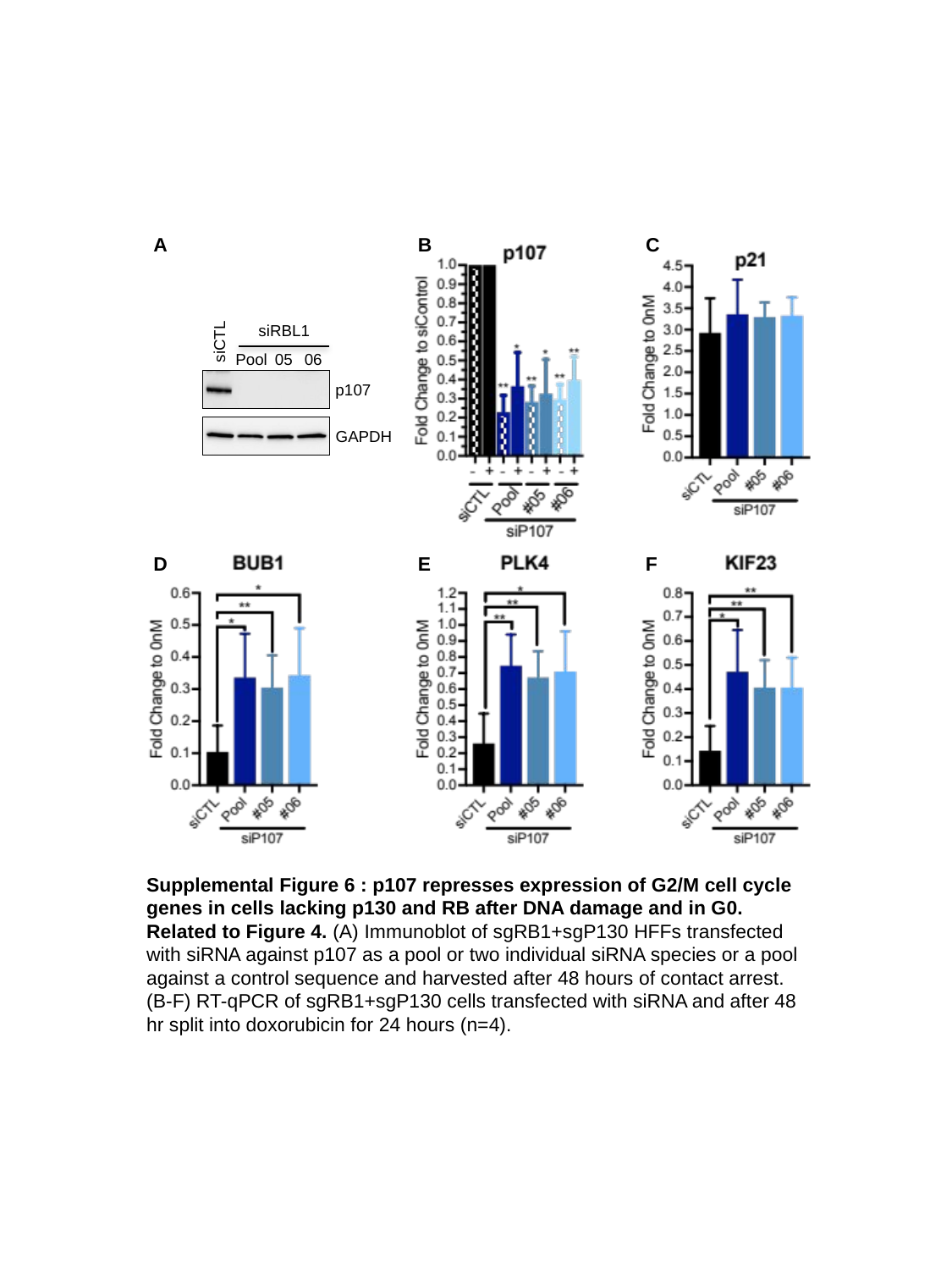

A
B
C
siRBL1
siCTL
Pool
05
06
p107
GAPDH
D
E
F
Supplemental Figure 6 : p107 represses expression of G2/M cell cycle genes in cells lacking p130 and RB after DNA damage and in G0. Related to Figure 4. (A) Immunoblot of sgRB1+sgP130 HFFs transfected with siRNA against p107 as a pool or two individual siRNA species or a pool against a control sequence and harvested after 48 hours of contact arrest. (B-F) RT-qPCR of sgRB1+sgP130 cells transfected with siRNA and after 48 hr split into doxorubicin for 24 hours (n=4).
